# Value of Continuous Hemofiltration in Patients with Severe Acute Pancreatitis at Onset: Single Centre Experience on 48 Patients

**DOI:** 10.3390/jcm14186647

**Published:** 2025-09-21

**Authors:** Paolina Saullo, Roberto Caronna, Alberto Maria Angelici, Valerio Rinaldi, Giovanni Liberatori, Andrea Mingoli, Piero Chirletti

**Affiliations:** 1 Department of Surgery, Policlinico Umberto I, Sapienza University of Rome, 00161 Rome, Italy; paolina.saullo@uniroma1.it (P.S.); roberto.caronna@uniroma1.it (R.C.); alberto.angelici@fondazione.uniroma1.it (A.M.A.); valerio.rinaldi@uniroma1.it (V.R.); andrea.mingoli@uniroma1.it (A.M.); 2 Department of Anesthesia and Intensive Care, Policlinico Casilino, 00169 Rome, Italy; giovanniliberatori@libero.it

**Keywords:** severe acute pancreatitis, cytokines, continuous veno-venous haemofiltration, oXiris filter, multiple organ dysfunction syndrome

## Abstract

**Background**: Severe acute pancreatitis (SAP) presents with Multiple Organ Dysfunction Syndrome (MODS) in ~15% of cases, accounting for ~35% of early deaths within 48 h. Major complications—shock, renal failure, and respiratory insufficiency—arise from an overwhelming systemic inflammatory response driven by markedly elevated pro-inflammatory cytokines. Massive release of IL-2, IL-6, and TNF-α underlies the systemic inflammatory response syndrome (SIRS). Continuous veno-venous hemofiltration (CVVH) with the oXiris filter, adsorbing endotoxins and cytokines, has been used in sepsis and applied early in SAP to reduce cytokine load and organ injury. **Aims**: To evaluate the efficacy and safety of early CVVH with the oXiris filter in modulating the systemic inflammatory response by removing toxic cytokines from the bloodstream in patients with SAP complicated by organ dysfunction and refractory sepsis. **Methods**: This single-centre, retrospective, observational study was conducted at a tertiary university hospital between 2000 and 2022. Forty-eight consecutive patients with SAP at onset, defined according to the 2012 Atlanta Classification, with an APACHE II score ≥ 19 and persistent organ dysfunction (>48 h), were included. All patients were unresponsive to initial intensive care within the first 24 h and underwent urgent laparotomy with extensive peritoneal lavage, pancreatic necrosectomy, and placement of multiple abdominal drains, followed by transfer to the intensive care unit. CVVH (Prismax system) with the oXiris filter was initiated within 12 h post-surgery. IL-6 and TNF-α were selected as inflammatory markers and measured in both serum and ultrafiltrate at baseline (0 h) and at 24, 48, 72, and 96 h. These measurements were correlated with clinical parameters and prognostic scores (APACHE II, SOFA). **Results**: Treatment was well tolerated in all patients. The 28-day survival rate was 97.9%. There was a significant time-dependent decrease in IL-6 (*p* = 0.019) and TNF-α (*p* = 0.008) concentrations in the ultrafiltrate, consistent with high early adsorption followed by a reduced cytokine burden, whereas serum levels showed a non-significant downward trend (IL-6 *p* = 0.08; TNF-α *p* = 0.310). The APACHE II score decreased from 23 postoperatively to 8 by the second week (−65.2%; *p* = 0.013), with a statistically significant correlation between cytokine reduction and clinical improvement. Adverse events were rare and manageable. **Conclusions**: Early CVVH with the oXiris filter in SAP, complicated by MODS and refractory sepsis, proved safe, well-tolerated, and potentially effective in reducing cytokine burden and improving prognostic indices. These findings support the hypothesis of a relevant immunomodulatory effect, warranting prospective controlled trials to confirm its true impact on survival and organ recovery.

## 1. Introduction

Severe acute pancreatitis (SAP) is a highly complex clinical condition affecting approximately 20–30% of patients with acute pancreatitis, with an in-hospital mortality rate of 15%, which may exceed 35% in the presence of infected necrosis or multiorgan failure (MODS) [1].

SAP has a characteristic biphasic course. In the early phase (first 1–2 weeks from onset), pathogenesis is dominated by a marked sterile systemic inflammatory response syndrome (SIRS), in which sepsis is rare. In severe SIRS, massive activation of pro-inflammatory mediators (TNF-α, IL-1β, IL-6) can cause endothelial damage, diffuse oedema, and early MODS involving the respiratory, cardiovascular, renal, and hepatic systems. Pancreatic necrosis, which may develop within the first 96 h, tends to evolve over the first two weeks; its extent is not necessarily correlated with the presence or severity of SIRS. However, in patients with severe early organ dysfunction, necrosis is frequently detected on computed tomography. Peripancreatic fluid collections are common in this phase.

In the late phase (>1–2 weeks), there is a shift from a pro-inflammatory response to a state of relative immunosuppression, favouring bacterial translocation due to impaired intestinal barrier function. This results in a high risk of infection of pancreatic necrotic areas and peripancreatic collections.

Mortality in SAP therefore shows two distinct temporal peaks: an early peak, associated with severe SIRS and MOF, and a late peak, attributable to infected necrosis complicated by sepsis [2,3,4].

The most widely used clinical tools for assessing disease severity include the APACHE II and SOFA prognostic scores, in line with the 2012 Atlanta Classification criteria, which define SAP as acute pancreatitis associated with persistent organ failure for more than 48 h [2,3,5].

Currently, SAP management is based on intensive support—including haemodynamic, ventilatory, and nutritional support—close monitoring of local and systemic complications, and surgical intervention in the most complex cases refractory to conservative therapy. However, there are no specific therapies capable of effectively modulating the systemic inflammatory response [6].

In this context, extracorporeal blood purification techniques have generated increasing interest. In particular, continuous veno-venous hemofiltration (CVVH), especially at high volume (HVHF), can remove inflammatory mediators of intermediate molecular weight (such as IL-6 and TNF-α), potentially reducing the cytokine storm and improving survival, although clinical data remain partial and heterogeneous [7].

The oXiris filter is a CRRT membrane designed to combine renal support and extracorporeal immunomodulation in a single device. Its structure is based on AN69 (acrylonitrile–metallyl sulfonate sodium copolymer), which is biocompatible and offers excellent permeability for convective/diffusive clearance; an internal surface layer of polyethyleneimine (PEI), a cationic polymer that, due to its positive charge, enhances electrostatic adsorption of endotoxins and pro-inflammatory cytokines; and a heparinised blood-contacting surface, reducing contact activation of coagulation and improving tolerability during prolonged sessions [8,9,10].

The result is a “four-in-one” cartridge that combines renal replacement, cytokine removal, endotoxin removal, and reduced surface thrombogenicity. Operationally, oXiris functions like a standard CRRT hemofilter, but, compared with conventional filters, it adds a direct adsorption component allowing rapid “debulking” of circulating mediators during hyperinflammatory phases. It can also be used in patients at higher bleeding risk, as it often allows minimal anticoagulation protocols [11], and is compatible with major dialysis platforms (e.g., Prismaflex/Prismax).

Adsorptive capacity is greatest in the first hours and progressively saturates; to maintain clinical efficacy, many centres replace the set every 12–24 h. During use, a gradual increase in transmembrane pressure (TMP) may occur. The average functional lifespan is similar, sometimes slightly shorter, than that of conventional filters. As with all adsorptive systems, the action is not completely selective: in addition to endotoxins and cytokines, beneficial molecules (such as certain drugs or nutrients) may be removed, requiring close clinical monitoring, electrolyte assessment—particularly phosphate—and adjustment of therapeutic dosages if necessary.

In septic settings, several studies have demonstrated the superiority of this approach over standard filters in reducing IL-6, TNF-α, IL-8, serum lactate levels, and vasopressor requirements [12,13,14].

The aim of the present study was therefore to evaluate the efficacy and safety of continuous hemofiltration with the oXiris filter in patients with severe acute pancreatitis complicated by organ dysfunction and septic status, analysing its impact on inflammatory and prognostic parameters.

## 2. Materials and Methods

### 2.1. Study Design and Clinical Setting

A retrospective, single-centre observational study was conducted at the Unit of General Surgery and Pancreatic Diseases, Policlinico Umberto I, Rome, a tertiary university hospital. The study included patients treated between January 2000 and July 2022. Patients were also stratified according to the treatment period (before and after 2010) to evaluate potential temporal effects. All patients provided written informed consent for the use of their clinical data for research purposes. Clinical procedures and data collection were conducted in accordance with the ethical principles outlined in the Declaration of Helsinki (2013) and current regulations for retrospective research. Given its retrospective nature and the absence of a control group, the analysis was descriptive and aimed to explore clinical outcomes, cytokine dynamics, and safety signals associated with early CVVH using the oXiris filter (Baxter International Inc., Deerfield, IL, USA).

### 2.2. Patient Selection Criteria

A total of 48 consecutive adult patients (age ≥ 18 years) with a diagnosis of severe acute pancreatitis (SAP), defined according to the 2012 revised Atlanta Classification as the presence of persistent organ failure for more than 48 h and an APACHE II score ≥ 19, were included. The APACHE II threshold of ≥19 was pre-specified in our institutional protocol to select a very high-risk SAP profile with persistent organ dysfunction and to improve clinical homogeneity for exploratory analyses; APACHE II was calculated at ICU admission (within 24 h), and the same threshold was applied uniformly throughout the study period. Inclusion required the presence of persistent multiorgan dysfunction (>48 h) unresponsive to conventional intensive treatment, with an indication for urgent surgical intervention due to infected pancreatic necrosis or abdominal compartment syndrome.

In the study cohort, the predominant aetiology of severe acute pancreatitis was biliary lithiasis, observed in 34 of 48 patients (70.8%). Alcohol-induced pancreatitis was identified in 8 patients (16.7%), whereas hypertriglyceridemia accounted for 3 cases (6.3%). In an additional 3 patients (6.3%), pancreatitis was attributed to asparaginase-related toxicity during treatment for acute lymphoblastic leukaemia (ALL).

Exclusion criteria were as follows: pregnancy, age < 18 years, chronic renal failure on replacement therapy, end-stage liver disease, known immunodeficiency, or refusal of consent.

### 2.3. Preoperative Assessment and Surgical Procedure

Upon hospital admission, all patients underwent a detailed medical history review, a physical examination, and a complete laboratory evaluation, including a complete blood count, pancreatic enzymes (amylase and lipase), inflammatory markers (CRP and procalcitonin), arterial blood gas analysis, serum creatinine, and electrolyte levels. Clinical severity was quantified using APACHE II and SOFA scores at admission and reassessed daily.

All patients underwent urgent exploratory laparotomy with opening of retroperitoneal spaces and debridement of pancreatic necrosis, thorough peritoneal lavage, and placement of multiple intra-peritoneal drains.

### 2.4. Hemofiltration with oXiris Filter

CVVH using a Prismax machine (Baxter International Inc., Deerfield, IL, USA) with an oXiris filter began within 12 h post-surgery. Main settings included the following:

- Filter: oXiris (AN69 membrane modified with polyethyleneimine coating and heparinised), with adsorption capacity for endotoxins and cytokines.
- Mode: continuous convective hemofiltration (pre-dilution).
- Blood flow rate: ≥75 mL/min (standard 180 mL/min).
- Ultrafiltration dose: 35 mL/kg/h, delivered with balanced replacement solution (PrismaSol) in pre-dilution.
- Anticoagulation: low-molecular-weight heparin continuous infusion (5–10 U/kg/h) or citrate.
- Filter replacement: every 24 h or earlier in the event of circuit clotting.
- Treatment duration: minimum 72 h, extended until haemodynamic stabilisation and reduction in inflammatory markers (mean 5 days, range 3–7 days).

Cytokines adsorbed (TNF-α, IL-6) were measured in the serum and ultrafiltrate at baseline and every 24 h thereafter.

### 2.5. Outcome Measures and Data Collection

Clinical and laboratory parameters were recorded at predetermined intervals (T0 = pre-CVVH; T24, T48, T72, T96 h). Recorded variables included:

- Demographic and clinical data: age, sex, aetiology of pancreatitis, and comorbidities.
- Inflammatory markers: leukocyte count, serum C-reactive protein (CRP), procalcitonin (PCT), TNF-α and IL-6 measured in serum, peritoneal lavage fluid, and CVVH ultrafiltrate.
- Haemodynamic parameters: heart rate, mean arterial pressure (MAP), lactate levels, pH, and base excess.
- Organ function indices: serum creatinine, PaO_2_/FiO_2_ ratio, intra-abdominal pressure.
- Intraoperative microbiological cultures from intra-abdominal collections.
- Prognostic scores: APACHE II and SOFA, calculated daily.
- Adverse events: hypotension, filter clotting, electrolyte disturbances.

The primary outcome was the change in TNF-α and IL-6 levels between T0 and T96 h. Secondary outcomes included changes in APACHE II and SOFA scores, haemodynamic parameters, and incidence of adverse events. Twenty-eight-day survival was recorded as a descriptive endpoint.

### 2.6. Statistical Analysis

Data were analysed using SPSS Statistics v.27 (IBM, Armonk, NY, USA). Continuous variables were expressed as mean ± standard deviation (SD) or median (interquartile range), depending on the distribution, which was assessed using the Shapiro–Wilk test. Categorical variables were reported as numbers and percentages. Comparisons between pre- and post-treatment values were performed using the paired Student’s t-test or Wilcoxon signed-rank test for non-parametric data. A *p*-value < 0.05 was considered statistically significant.

### 2.7. Ethical Considerations

The study did not require formal approval from the Ethics Committee as it was a retrospective observational analysis. All patients provided informed consent for the use of clinical data. Data were collected and processed in anonymised and aggregated form to ensure confidentiality and privacy protection.

## 3. Results

### 3.1. Population and Clinical Characteristics

Between January 2000 and July 2022, of 135 patients hospitalised for acute pancreatitis, 48 (35.6%) presented with severe acute pancreatitis (SAP) with an APACHE II score ≥ 19 and persistent multiorgan failure unresponsive to conventional intensive care. The cohort included 30 women (62.5%) and 18 men (37.5%), with a mean age of 60.4 ± 18 years.

At the time of surgical assessment, 39 patients (81.3%) had abdominal compartment syndrome and 9 (18.8%) presented with septic shock refractory to intensive care. All underwent urgent laparotomy with thorough peritoneal lavage, pancreatic necrosectomy, and placement of multiple abdominal drains, followed by transfer to the intensive care unit and initiation of CVVH with the oXiris (AN69-based) filter (surface area 1.2 m^2^) within 12 h postoperatively. Treatment parameters included a blood flow rate of 75 mL/min, an ultrafiltration dose of 35 mL/kg/h (pre-dilution, PrismaSol), anticoagulation with low-molecular-weight heparin, and filter replacement every 24 h. Mean CVVH duration was 6 days (range 3–8) (Table 1). To explore potential temporal effects associated with the long recruitment period, patients were stratified according to treatment period (before vs. after 2010). No relevant differences were observed between the two subgroups in terms of baseline severity (APACHE II), cytokine kinetics, or 28-day survival. Although the limited sample size did not allow for definitive statistical testing, the consistency of findings across both time frames suggests that temporal changes in general SAP management did not substantially affect the outcomes observed in this cohort.

### 3.2. Primary and Secondary Outcomes

#### 3.2.1. Tolerability and Survival

All patients tolerated the treatment well. Overall, in-hospital survival was 97.9% (47/48), with a single death (2.1%) due to septic shock from Acinetobacter baumannii. The only surgical complication reported was an enteric fistula, managed conservatively. No major bleeding or ischaemic complications occurred.

#### 3.2.2. Hospital Stay

Mean total hospital stay was 28.5 ± 19 days, with a mean intensive care unit stay of 13.3 ± 11 days. Intraoperative cultures yielded *Enterococcus* spp. in 30 patients (62.5%), Escherichia coli in 10 (20.8%), Pseudomonas aeruginosa in 5 (10.4%), and Acinetobacter baumannii in 1 (2.1%); cultures were sterile in 2 patients (4.2%).

#### 3.2.3. Changes in Inflammatory Biomarkers and Clinical Scores

At baseline (T0), serum concentrations of IL-6 (40.8 ± 8 pg/mL) and TNF-α (13.5 ± 2.1 pg/mL) were elevated in all patients. During CVVH treatment with the oXiris membrane, both cytokines were also detected in the ultrafiltrate, initially at high levels (IL-6: 15.3 ± 5 pg/mL; TNF-α: 8.0 ± 2.0 pg/mL at intervention) and subsequently showing a progressive decline over time (IL-6: 7.3 ± 2 pg/mL and TNF-α: 4.6 ± 1.4 pg/mL at POD X), indicating effective removal through convection and adsorption.

**Figure 1:**
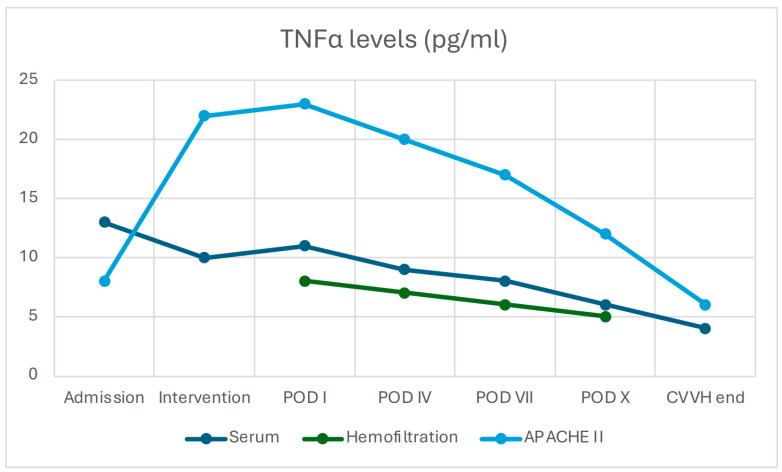
TNF-α levels in serum and hemofiltration effluent and APACHE II scores during CVVH (*n* = 48). Data are displayed at predefined time points (admission, intervention, POD I–X, end of CVVH) as mean ± SD. Serum TNF-α showed an overall downward trend (from 13.5 ± 2.1 to 5.5 ± 1.5 pg/mL) that did not reach statistical significance (*p* = 0.31), whereas effluent TNF-α declined progressively (from 8.0 ± 2.0 to 4.6 ± 1.4 pg/mL), consistent with extracorporeal removal. Spearman’s correlations demonstrated a significant association between effluent TNF-α and APACHE II (*p* < 0.05), while no significant correlation was observed for serum TNF-α (n.s.). (APACHE: Acute Physiology and Chronic Health Evaluation, POD: Post Operative Day).

**Figure 2:**
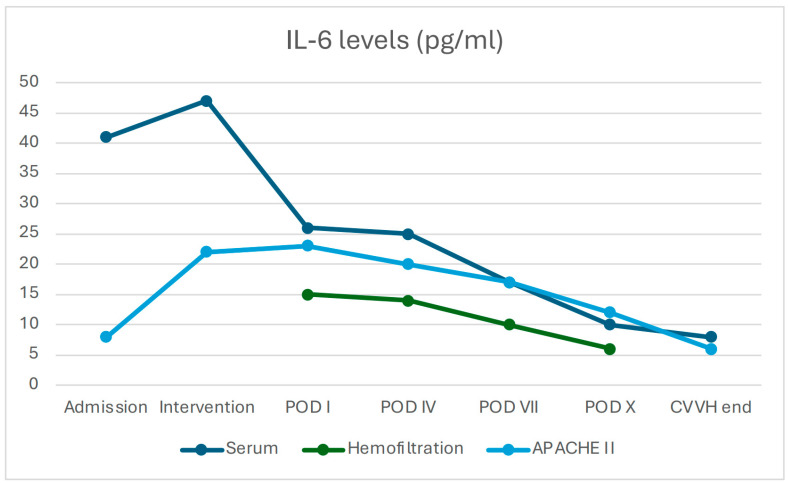
Correlation between IL-6 levels across compartments (serum and hemofiltration effluent) and APACHE II scores during CVVH (*n* = 48). Data are shown at predefined time points (admission, intervention, POD I–X, end of CVVH). Serum IL-6 displayed an overall downward trend (from 40.8 ± 8 to 10 ± 3 pg/mL) without reaching statistical significance (*p* = 0.08), whereas effluent IL-6 declined progressively (from 15.3 ± 5 to 7.3 ± 2 pg/mL), consistent with extracorporeal removal. APACHE II scores decreased significantly over the same period (*p* < 0.05). Correlations between IL-6 measures and APACHE II were evaluated using Spearman’s rank test. (APACHE: Acute Physiology and Chronic Health Evaluation, POD: Post Operative Day).

In parallel, serum concentrations of both IL-6 and TNF-α exhibited a clear downward trend (IL-6 decreased from 40.8 ± 8 to 10 ± 3 pg/mL, *p* = 0.08; TNF-α from 13.5 ± 2.1 to 5.5 ± 1.5 pg/mL, *p* = 0.310), although without reaching statistical significance, likely due to the ongoing endogenous production of pro-inflammatory mediators (Figure 1 and Figure 2).

#### 3.2.4. Filter Performance

The consistently high cytokine concentration in the hemofiltration effluent confirmed the AN69 filter’s efficacy in adsorbing pro-inflammatory mediators. Paired data analysis demonstrated a significant correlation between cytokine reduction and clinical improvement (decrease in APACHE II score).

#### 3.2.5. Prognostic Scores

The APACHE II score was 23 on the first postoperative day. It decreased to 18.6 by day 4 (−19.1%) and reached 8 by the second week (−65.2%; *p* = 0.013). The reduction in APACHE II scores was significantly correlated with decreases in serum IL-6 and TNF-α levels (non-parametric correlation, *p* < 0.05).

#### 3.2.6. Safety Profile

CVVH with the oXiris filter was overall well tolerated in our cohort. No cases of major bleeding or ischemic complications were observed. Filter clotting did not represent a clinically relevant issue, as circuits were routinely replaced every 24 h and no premature discontinuation of treatment was required. Electrolyte disturbances were limited to hypophosphatemia in 2 patients (4.2%), which was promptly corrected with supplementation. One patient (2.1%) developed a febrile episode related to central venous catheter infection, which resolved after catheter removal. No red blood cell transfusions were directly attributable to CVVH. Importantly, no adverse event resulted in a permanent interruption of therapy (Table 2).

## 4. Discussion

In agreement with prior studies on CVVH in SAP [2,15,16,17,18,19,20,21,22] and with the evidence supporting the adsorptive oXiris membrane in sepsis [7,8,9,10,11,12,23], our study demonstrates that early use of continuous veno-venous hemofiltration (CVVH) with the oXiris filter in patients with severe acute pancreatitis (SAP) complicated by organ dysfunction and sepsis refractory to intensive care is associated with a high short-term survival rate (97.9%), a significant reduction in APACHE II score, and a marked decrease in serum pro-inflammatory cytokines (IL-6, TNF-α). The progressive increase in cytokine concentration in the dialysis effluent, coupled with their reduction in the blood circuit and systemically, suggests effective extracorporeal removal of inflammatory mediators, supporting the hypothesis of a genuine immunomodulatory effect of the treatment. These results are consistent with international literature.

Several studies have addressed the use of hemofiltration with oXiris in severe sepsis, a pathophysiological setting closely related to severe SAP. These studies have shown that the oXiris filter removes endotoxins and cytokines more effectively than standard filters, with haemodynamic benefits and lactate reduction [7,8,9,14,23]. Observational studies [12] confirm improvements in SOFA score, haemodynamic stability, and reduced vasopressor requirements within the first 24 h. However, evidence remains heterogeneous across studies and systematic reviews, and not all analyses demonstrate a survival advantage [15,16,19].

Evidence specific to SAP is more limited but promising. The meta-analysis by Guo et al. [17], involving over 1200 patients, demonstrated that CVVH reduces mortality, APACHE II score, and inflammatory markers compared to standard treatment, particularly when initiated within a few days of onset. Huang et al. [16] confirmed that high-volume hemofiltration reduces early mortality and infections, although it does not significantly affect the incidence of MODS or overall hospital stay. Additional evidence comes from studies in hyperlipidaemic or septic pancreatitis, where the combination of high-volume hemofiltration and haemoperfusion significantly reduced APACHE II and SOFA scores and improved haemodynamic stability [24,25].

Cui et al. showed, in patients with SAP complicated by ARDS, not only a significant reduction in IL-6 and TNF-α after just 24 h of treatment, but also an improvement in ARDS severity and blood oxygenation [26]. Some studies also suggest a potential advantage of early CVVH in reducing the incidence of pancreatic pseudocysts [27].

Clinical data also indicate that timing is crucial: Jiang et al. [18] demonstrated that initiation within 48 h, particularly with high filtration volumes, improves survival (94% vs. 68%) and more effectively reduces TNF-α, IL-1β, and IL-6 compared to delayed or standard-volume CVVH. Xu et al. [5] found that early CVVH in patients with SAP and abdominal compartment syndrome rapidly reduces intra-abdominal pressure, improves organ function, and decreases mortality.

Although literature specific to SAP remains limited, available evidence is encouraging, showing substantial reductions in inflammatory biomarkers, improvement in haemodynamic status, and better metabolic parameters within 24 h of initiating CRRT [17,19,20,22,28]. Regarding the specific use of oXiris in SAP, only one recent case report [29] exists, describing complete clinical recovery following combined CVVH with the oXiris filter in a paediatric patient with drug-induced severe acute pancreatitis and multiorgan failure. Our results align with these findings, documenting the filter’s efficacy in adsorbing inflammatory cytokines and contributing to early clinical stabilisation.

### 4.1. Pathophysiological Mechanisms Involved

The beneficial effect of hemofiltration in SAP can be attributed to a dual mechanism: the convective removal of intermediate-molecular-weight molecules (such as IL-6, IL-1β, TNF-α) and, in the case of adsorptive filters like oXiris, selective elimination of endotoxins and cytokines via surface interactions. This strategy helps attenuate the hyperactive systemic inflammatory response syndrome (SIRS) and prevents progression to multiorgan dysfunction syndrome (MODS). Our study reinforces these hypotheses, demonstrating a direct correlation between cytokine reduction and improvement in APACHE II scores.

Overall, available data suggest that the efficacy of hemofiltration in SAP depends on key variables: timing of initiation, filtration volume, treatment duration, and type of filter used. In our patients, the choice to initiate CVVH early, employ a high-adsorptive-capacity filter, and maintain an adequate convective dose may have contributed to the positive outcomes observed.

### 4.2. Limitations

This study was retrospective, single-centre, and conducted without a parallel control group, which limits the ability to establish causal relationships between CVVH with the oXiris filter and the observed outcomes. Nonetheless, the consistent improvements in clinical parameters, cytokine profiles, and prognostic scores provide clinically relevant insights and support the rationale for prospective multicentre studies. In addition, a stratified comparison of patients treated before and after 2010 did not show relevant differences in baseline severity, cytokine dynamics, or short-term outcomes. Although the small sample size precludes definitive conclusions, these findings suggest that temporal changes in SAP management are unlikely to have substantially influenced the results.

## 5. Conclusions

Early initiation of continuous veno-venous hemofiltration (CVVH) with the adsorptive AN69-based oXiris filter in patients with severe acute pancreatitis (SAP) and organ dysfunction was associated, in our experience, with significant clinical benefits. We observed a marked reduction in pro-inflammatory cytokines (IL-6 and TNF-α) both in serum—although not statistically significant—and, significantly, in the hemofiltration effluent, together with a significant improvement in APACHE II scores and a very high short-term survival rate (97.9%). The treatment also demonstrated a favourable safety profile, with rare and manageable complications.

These findings support the hypothesis of a tangible immunomodulatory effect of CVVH with the oXiris filter, potentially capable of attenuating the “cytokine storm” characteristic of the most severe forms of SAP. Nevertheless, serum IL-6 and TNF-α levels showed only a non-significant downward trend, which warrants caution when inferring a direct systemic effect. These observations, although suggestive of an extracorporeal immunomodulatory effect, do not establish a causal benefit or clarify any impact on mortality or organ recovery.

Prospective, multicentre, randomised trials are therefore needed to validate these preliminary findings, assess their effects on mortality, organ failure resolution, and health-related quality of life, and define the optimal timing, filtration dose, and treatment duration for potential clinical benefit.

## Figures and Tables

**Table 1:** Patient demographics, aetiology of pancreatitis and comorbidities.

Sex, *n* (%)	F: 30 (62.5) M: 18 (37.5)
Age, years, mean ± SD	60.4 ± 18
Aetiology, *n* (%)	Biliary lithiasis, 34 (70.8) Alcoholic pancreatitis, 8 (16.7) Hypertriglyceridemia, 3 (6.3) Asparaginase-induced pancreatitis (ALL), 3 (6.3)
Comorbidities, *n* (%)	Arterial hypertension, 21 (43.8) COPD, 4 (8.3) CKD, 6 (12.5) DM, 9 (18.8) Obesity (BMI > 30), 17 (35.4)

F: Female, M: Male, SD: Standard Deviation, COPD: Chronic Obstructive Pulmonary Disease, CKD: Chronic Kidney Disease, DM: Diabetes Mellitus, ALL: Acute Lymphoblastic Leukaemia.

**Table 2:** Primary and Secondary Outcomes.

Primary Outcomes	
Tolerability, *n* (%)	48 (100)
Survival, *n* (%)	47 (97.9)
Secondary Outcomes	
Surgical Complications, *n* (%)	Enteric fistula, 1 (2.1) Major bleeding– ischemic complications–
Hospital Stay, days, mean ± SD	28.5 ± 19
Microorganisms isolated from intraoperative cultures, *n* (%)	*Enterococcus* spp., 30 (62.5) *Escherichia Coli*, 10 (20.8) *Pseudomonas Aeruginosa*, 5 (10.4) *Acinetobacter baumannii*, 1 (2.1) *Sterile cultures*, 2 (4.2)
ICU stay, days, mean ± SD	13.3 ± 11
CVVH adverse events, *n* (%)	Fever from CVC, 1 (2.1) Hypophosphatemia, 2 (4.2)

ICU: Intensive care unit, CVVH: Continuous Veno-Venous Hemofiltration, CVC: Central Venous Catheter.

## Data Availability

The raw data supporting the conclusions of this article will be made available by the authors on request.

## Abbreviations

The following abbreviations are used in this manuscript:

SAP	Severe acute pancreatitis
MODS	Multiple Organ Dysfunction Syndrome
SIRS	Systemic Inflammatory Response Syndrome
CVVH	Continuous Veno-Venous Hemofiltration
HVHF	High-Volume Hemofiltration
CRRT	Continuous Renal Replacement Therapy
AN69	Acrylonitrile–metallyl sulfonate sodium copolymer 69
PEI	Polyethyleneimine
TMP	Transmembrane Pressure
APACHE II	Acute Physiology and Chronic Health Evaluation II
SOFA	Sequential Organ Failure Assessment
ICU	Intensive Care Unit
POD	Post-Operative Day
CVC	Central Venous Catheter
COPD	Chronic Obstructive Pulmonary Disease
CKD	Chronic Kidney Disease
DM	Diabetes Mellitus
ALL	Acute Lymphoblastic Leukaemia
CRP	C-Reactive Protein
PCT	Procalcitonin
MAP	Mean Arterial Pressure
ARDS	Acute Respiratory Distress Syndrome
